# Neglected Funding for Vector-Borne Diseases: A Near Miss This Time, a Possible Disaster the Next Time

**DOI:** 10.1371/journal.pntd.0000847

**Published:** 2010-10-26

**Authors:** A. Desirée LaBeaud, Serap Aksoy

**Affiliations:** 1 Center for Immunobiology and Vaccine Development, Children's Hospital Oakland Research Institute, Oakland, California, United States of America; 2 Epidemiology and Public Health, Yale School of Public Health, New Haven, Connecticut, United States of America

Vector-borne diseases (VBDs) are some of the world's most common and devastating maladies. Despite this truth, the United States government had decided to drastically cut funding for the Division of Vector-Borne Infectious Diseases (DVBID) program of the Centers for Disease Control and Prevention (CDC) in the Fiscal Year 2011 Labor, Health and Human Services and Education appropriations bill [1]. Nearly US$27 million had been removed from the DVBID financial plan in the President's FY 2011 budget, slashing DVBID funding from US$39 million to US$12 million. Although the program is minimally supported by other agencies, this 70% funding cut would have virtually eliminated the DVBID program.

Many organizations, including the American Society of Tropical Medicine and Hygiene, the Infectious Disease Society of America, the American Society for Microbiology, the American Red Cross, and others, appealed to restore funding for the DVBID program. At the end of July, the Senate restored this funding completely at US$26.7 million in their version of the FY 2011 appropriations bill, and currently we are waiting for the House to reveal its version. It is our hope that after reconciling the two bills, DVBID program funding will be completely restored.

VBDs are easily targeted for cutbacks in public health funding because their incidence, prevalence, morbidity, and associated mortality are routinely underestimated. Their impact is not adequately captured by current disease burden assessments, and therefore VBDs are often not included in top-level discussions of disease-control priorities [2]–[5]. We feel strongly that funding cuts are short-sighted and that continued surveillance and control are worth the investment. In the absence of a proactive surveillance system that provides valid national and regional data on VBD transmission, outbreak epidemiology would likely be done by “official denial”, and subsequent public health responses would likely be poorly managed and of limited effectiveness [6], [7].

## Burden of Vector-Borne Diseases

VBDs of major public health importance include a wide variety of bacterial, parasitic, and viral infections that are spread by blood-feeding arthropods. In North America, prominent examples of VBDs include West Nile virus (WNV), Lyme disease, and dengue virus (Table 1). In the United States, the most common tick-borne infection is Lyme disease, which results in extensive health care costs and productivity losses. In 2008 alone, there were 35,198 cases of Lyme disease reported in the US.

**Table 1:** Cases of Vector-Borne Diseases by Pathogen Group in the US Reported to the CDC [30].

VBD	2000	2001	2002	2003	2004	2005	2006	2007	2008
Viruses	140	393	2,074	12,891	1,272	3,114	4,384	3,748	1,435
Bacteria	18,373	15,530	18,507	21,421	21,654	25,403	22,331	29,809	37,887
Parasites	1,560	1,414	1,199	1,278	1,458	1,494	1,474	1,408	1,255
Total	20,073	17,337	21,780	35,590	24,384	30,011	28,189	34,965	40,577

The virus category includes California serogroup, Eastern equine, Western equine, St. Louis, West Nile, and La Crosse encephalitis and non-neuroinvasive (2003: 9,862; 2005: 1,704; 2006: 2,779; 2008: 679) cases when reported. The bacteria category includes Lyme disease (confirmed and probable), tularemia, Rocky Mountain spotted fever, and plague cases. The parasite category reflects reported malaria cases.

Similarly, arthropod-borne viral infections, or arboviral infections, are common causes of disabling fever syndromes worldwide. These often progress to complications such as encephalitis or hemorrhagic fever, which result in severe long-term physical and cognitive impairment, or in early death [8], [9]. Between 2002 and 2008, 28,812 cases of West Nile disease were reported in the US. The majority of these (21,277) were neuroinvasive disease, a statistic that reflects the serious underreporting of mild cases of WNV–associated disease. Several other menacing arboviruses are considered to be “emerging pathogens”, based on their recent geographic spread and their increasing impact on susceptible human populations [10]–[19]. As an example, dengue virus infections, once rare, are now estimated to cause >50 million clinical cases per year following a resurgence in Asia and the virus's respread through Central and South America [20]. The US is now threatened by the potential emergence of public health threats such as Rift Valley fever and chikungunya virus, which could easily establish themselves within our ecosystems. The recent emergence of dengue in Texas (2005) and Florida (2009–2010) demonstrates our continuing vulnerability to such arboviral pathogens [21].

## Multi-Faceted Roles of the DVBID

The Vector-Borne Infectious Diseases program supports work on agents such as WNV, plague, tularemia, yellow fever, Lyme disease, dengue fever, Japanese encephalitis, and other arboviral encephalitides. The mission of the division is to 1) develop and maintain effective surveillance for vector-borne viral and bacterial agents and their arthropod vectors; 2) conduct field and laboratory research and epidemic aid investigations; 3) define disease etiology, ecology, and pathogenesis in order to develop improved methods and strategies for disease diagnosis, surveillance, prevention, and control; 4) provide diagnostic reference and epidemiologic consultation, on request, to state and local health departments, other components of the CDC, other federal agencies, and national and international health organizations; and 5) provide intramural and extramural technical expertise and assistance in professional training activities [22]. In addition, the program maintains vital expertise for other vector-borne infectious diseases that occur only sporadically or in periodic epidemics. The DVBID program integrates local, state, and national labs to create and maintain national and regional data that lead to quick identification of the causes of outbreaks and rapid, effective response.

Not only does DVBID provide state, national, and international support for surveillance of threatening VBDs, it also creates opportunities for training, cutting edge research, and new control methodologies. The program currently allocates about half of its budget to state epidemiology programs to track and control VBDs. The surveillance that DVBID supports in local and state health departments allows for the estimation of prevalence and incidence data that funnels to other crucial programs, such as safety testing of our blood supply. In addition to tracking diseases that are currently circulating, the DVBID program also provides the infrastructure and expertise to identify emerging or new pathogens. As such, it is part of our first line of defense against the accidental or intentional introduction of biodefense pathogens such as plague, tularemia, typhus, dengue, and many other viral hemorrhagic fevers and encephalitides.

## Consequences of Neglected Surveillance

Put simply, proper surveillance prevents illness in humans and animals. Elimination of funding for the DVBID program would jeopardize our nation's security and welfare. Funding cuts would dismantle the current system and erase the effects of the US$200 million and human capital that has already been invested in the DVBID program. The CDC estimates this cut could lead to job losses for 100 state and 24 CDC employees and devastate the CDC's Dengue Branch in San Juan, Puerto Rico, which also acts as a World Health Organization (WHO) reference center. Not only would jobs be lost, but strategic partnerships between local, state, and federal partners would also dwindle. Once expertise and technical resources are lost, they cannot be quickly recovered [6]. Neither can existing “tacit knowledge” be quickly regained on how to provide effective vector control. The National Institutes of Health does not routinely support research in surveillance; therefore, without CDC's activities in this vital task, it would go undone. As a result, the American public would become more vulnerable to some of the most threatening emerging and reemerging diseases of our time.

The proposed cut in this program would also lead to significant delays in outbreak response and the identification of new pathogens. This, in turn, would result in unnecessary costs and patient harm when VBD outbreaks occur. In this issue of *PLoS Neglected Tropical Diseases*, Vazquez-Prokopec and colleagues show us that the costs of surveillance are far lower than the costs of delayed outbreak response, even without considering the costs of infection-related deaths and disability [7]. Because many VBDs are also zoonoses, cuts in control would also place animal health in jeopardy. For example, if Rift Valley fever virus is imported to the US, livestock, wildlife, and humans will all be affected. The monetary cuts would lead to irreplaceable cuts in expertise and human capital and lead to delayed recognition of new VBDs. Research in new diagnostics, prevention, and control efforts would also be decimated, undoubtedly with major global repercussions.

## Simple Solutions for Complex Diseases

As new diseases emerge and old diseases reemerge, we believe that the government should actually increase the budget for the DVBID. The old dictum states that an ounce of prevention is worth a pound of cure. In no other part of the nation's health system is this truer than with regard to VBDs. No treatments exist for many of these infections, and vaccines are under development for only a few VBDs, such as WNV and dengue. Therefore, surveillance and vector control remain our only practical tools for prevention of disease. Continued development of vaccines for VBDs should remain a priority. Evidence suggests that a recent surge in global yellow fever cases has been due to the decline in mosquito control and yellow fever virus vaccination efforts. This fact highlights the tenuous hold we maintain over these infections, and draws attention to the immediate increase in disease burden that should be expected from any lack of persistent focus on surveillance and control [23], [24].

Control is the only way to stop these infections from emerging, and in order to control these infections, we need to know where they are circulating. In the past five years, dengue virus has spread along the Texas and Florida borders, and is expanding rapidly in Puerto Rico. Meanwhile, WNV continues to cause severe neurological disease across America. Other persistent VBDs, including La Crosse virus in the Midwest and Eastern equine encephalitis in Florida, Massachusetts, Michigan, Georgia, and Louisiana, have caused significant outbreaks this year. Although the numbers appear small by national health statistics, they are kept small by the persistence and focus of the DVBID surveillance program (Table 1). Substantial economic losses and health care disruption can result from severe arboviral outbreaks [7], [25]. Any one of these infections could result in a major, multi-state outbreak if weather conditions were favorable and control wasn't immediate and effective. To appropriately address VBDs, the US requires an energetic infrastructure for detection, diagnosis, response, and prevention at the national, state, and local levels.

We have witnessed the devastation these infections can inflict on healthy children in the US and abroad. As the world becomes increasingly networked through globalization, and as climate change continues to modify vector distribution and abundance worldwide, infections that were once contained in remote tropical locations, such as Japanese encephalitis and Rift Valley fever, are likely to spread to new areas. Just as WNV emerged in the US in 1999, other arboviral pathogens will escape control to infect large susceptible populations [12], [26]–[29]. Continuing public health surveillance for VBDs and continuing vector control (outside the standard health care delivery systems) are crucial to preventing these diseases and the morbidity and mortality that they cause. The benefits of continued (and improved) surveillance for VBDs are undeniable.
